# Access to and Use of Point-of-Care Ultrasound in the Emergency Department

**DOI:** 10.5811/westjem.2015.7.27216

**Published:** 2015-10-20

**Authors:** Jason L. Sanders, Vicki E. Noble, Ali S. Raja, Ashley F. Sullivan, Carlos A. Camargo

**Affiliations:** Massachusetts General Hospital, Department of Emergency Medicine, Boston, Massachusetts

## Abstract

**Introduction:**

Growing evidence supports emergency physician (EP)-performed point-of-care ultrasound (PoC US). However, there is a utilization gap between academic emergency departments (ED) and other emergency settings. We elucidated barriers to PoC US use in a multistate sample of predominantly non-academic EDs to inform future strategies to increase PoC US utilization, particularly in non-academic centers.

**Methods:**

In 2010, we surveyed ED directors in five states (Arkansas, Hawaii, Minnesota, Vermont, and Wyoming; n=242 EDs) about general ED characteristics. In four states we determined barriers to PoC US use, proportion of EPs using PoC US, use privileges, and whether EPs can bill for PoC US.

**Results:**

Response rates were >80% in each state. Overall, 47% of EDs reported PoC US availability. Availability varied by state, from 34% of EDs in Arkansas to 85% in Vermont. Availability was associated with higher ED visit volume, and percent of EPs who were board certified/board eligible in emergency medicine. The greatest barriers to use were limited training (70%), expense (39%), and limited need (perceived or real) (32%). When PoC US was used by EPs, 50% used it daily, 44% had privileges not requiring radiology confirmation, and 34% could bill separately for PoC US. Only 12% of EPs used it ≥80% of the time when placing central venous lines.

**Conclusion:**

Only 47% of EDs in our five-state sample of predominantly non-academic EDs had PoC US immediately available. When available, the greatest barriers to use were limited training, expense, and limited need. Recent educational and technical advancements may help overcome these barriers.

## INTRODUCTION

As ultrasound technology improves, and as pressures on emergency physicians (EPs) grow to see ever more patients quickly and cost effectively, there has been a surge in literature demonstrating that point-of-care ultrasound (PoC US) can decrease cost, 1 reduce need for additional diagnostic testing, 2 improve patient throughput 3 and patient satisfaction, 4 and may reduce need for imaging with ionizing radiation. 5 Accordingly, PoC US image acquisition and interpretation is now a core competency for emergency medicine residency training.

Despite this growing evidence base and improved training efforts, previous surveys of PoC US have demonstrated a utilization gap, most notably between rural and urban emergency departments (ED), low and high volume EDs, and EDs with a lower proportions of emergency medicine board certified/board eligible (EM BC/BE) EPs vs. EDs with more EM BC/BE EPs. 6 These distinctions are important because most individuals do not receive emergency care at an academic center. 7 Building on previous work surveying PoC US at diverse practice sites across the United States, 6 we performed a more detailed survey to study PoC US utilization and determine specific barriers to utilization.

## METHODS

### Identifying Emergency Departments: NEDI-USA Survey

Our data are drawn from EDs in five diverse states: Arkansas, Hawaii, Minnesota, Vermont, and Wyoming. These states were chosen due to their geographic diversity and distribution of EDs, which include many non-academic EDs (only 4% of EDs in these states are a part of hospitals in the Council of Teaching Hospitals) and many EDs with lower patient volume, which are often not surveyed in ED operations research. To identify eligible EDs in the five states, in 2010 we used the 2009 version of the National Emergency Department Inventory (NEDI)-USA database, which provided a comprehensive list of all nonfederal U.S. hospitals with EDs. The methods for creation of the NEDI-USA database have been previously described. 8 Emergency Medicine Network (Boston, MA) staff compile NEDI-USA through original data collection and integration of information from a variety of sources (e.g., Intercontinental Marketing Services Health Hospital Market Profiling Solution, American Hospital Association Annual Survey Database, Flex Monitoring Team, and Association of American Medical Colleges). EDs were defined as emergency care facilities open 24/7, and available for use by the general public. We excluded federal hospitals (e.g., Veterans Affairs, Indian Health Service, and military hospitals), specialty hospitals (e.g., psychiatric hospitals), and college infirmaries. NEDI-USA was approved by the institutional review board of Massachusetts General Hospital. Each state investigator’s institutional review board approved the study with a waiver of written informed consent. Responses were based on respondent estimates for the year 2009. NEDI-USA surveys were mailed to ED directors, with up to two follow-up mailings sent to non-respondents. If we received an incomplete or no response, mailed surveys were followed by telephone contact. We used a mailed survey rather than an online survey because we have found that many ED directors of smaller, rural EDs prefer mailed surveys and their participation is critical to the generalizability of data collected as part of the NEDI project.

### Measuring Emergency Department Characteristics: NEDI-State Survey

After identifying eligible EDs with the NEDI-USA survey, we obtained detailed information on EDs with the NEDI-State survey. The NEDI-State survey is rooted in measuring basic, real world operational characteristic of the ED (see Supplementary Survey for questions, such as, “Is your emergency department open 365 days per year?”). The survey was initially developed by investigators within the Emergency Medicine Network. Following this phase, the survey was sent to multiple independent EP reviewers from across the United States to iteratively improve the survey and establish greater face validity. Physician reviewers were drawn from a variety of settings including members of one or more American College of Emergency Physicians chapter boards. The completed survey has been deployed successfully in 2006 in Massachusetts 9 and in 2009 in four states, 6 with >80% response rate in every state.

### Ultrasound Variables

NEDI-State surveys included questions on basic ED characteristics, staffing, electronic resources, PoC US, timing of consultations, tests, and transfers, and ED crowding. The two key survey questions on PoC US were, “Is bedside ultrasound immediately available in the ED?” and, “In your ED, do the emergency physicians (not radiologists, cardiologists, etc.) use bedside ultrasound for clinical care?” EDs in four of the states (Arkansas, Hawaii, Vermont, and Wyoming) were asked additional questions to determine characteristics of PoC US use by EPs. In particular, EDs not reporting use of PoC US were asked to identify barriers to use.

### Additional ED Variables

We categorized ED location as urban or rural (adjacent to urban or not adjacent to urban) using county-based 2003 urban influence codes (www.usda.gov). ED volume was represented by the number of patients seen per hour, calculated from annual visit volume. We used hospital admission rate as a surrogate for ED acuity. Patient population was categorized using the percent of patients uninsured or who self-pay. Characteristics of physician staffing included the total number of EP full-time equivalents (FTEs) and the proportion of physicians who were BC/BE EPs by the American Board of Emergency Medicine, American Osteopathic Board of Emergency Medicine, or the American Board of Pediatrics (Pediatric Emergency Medicine).

### Statistical Analysis

We used descriptive statistics to summarize data on the overall sample and by presence or absence of PoC US. Bivariate associations between PoC US use and ED characteristics were calculated using chi-square and Fisher’s exact tests. We used multivariable logistic regression to determine the independent odds of PoC US availability by each ED characteristic adjusted for other characteristics in the model. Two-tailed P-values were calculated, with P<0.05 representing statistical significance. Summary statistics were also used to display the proportion of EDs reporting specific barriers to PoC US and PoC US use patterns. We performed statistical analysis using SAS 9.3 (SAS Institute, Cary, NC).

## RESULTS

From the NEDI-USA survey we identified 271 EDs in the five states. Overall, 242 of 271 sites provided data for analysis (89% response rate) from the NEDI-State survey, with >80% response rate in every state. Among the respondents, 201 provided complete information on PoC US (74%). Response rates for availability of PoC US were equivalent across urban/rural status, admission rate, patient insurance status, number of physician FTEs, and proportion of physicians that were EM BC/BE EPs; response rates were lower among EDs with lower visit volume and across states (data not shown).

In unadjusted analyses, PoC US availability varied among states and was higher in urban EDs, higher volume EDs, higher acuity EDs, EDs with more physician staffing, and EDs with a higher proportion of EM BC/BE EPs (Table 1). PoC US availability was not associated with patient insurance status. In multivariable logistic models adjusting for all characteristics simultaneously, each state had markedly different odds of PoC US availability compared to Arkansas: Hawaii OR=5.2, 95% confidence interval [1.03–26.6]; Minnesota OR=6.7, [2.3–19.7]; Vermont OR=15.4, [2.0–121.3]; Wyoming OR=10.2, [2.3–45.0]. PoC US was more likely to be available in EDs with higher visit volume (≥3 patients per hour vs. <1 patient per hour, OR=9.9, [1.9–51.6]) and more EM BC/BE physicians as a percent of physicians staffed in the ED (≥80% vs. 0% to less than 20%, OR=4.3, [1.5–12.2]). PoC US availability did not differ by urban/rural status, admission rate, number of physician FTEs, or insurance status.

In our four-state sample (123 total EDs) with more detailed information on PoC US, 52% of sites had PoC US available in the ED. At 43% of sites, EPs used PoC US for care (Table 2). The most common reason for PoC US being unavailable or not used by EPs was limited training (70%), PoC US being too expensive (39%), or having limited need (perceived or real) (32%). Few sites (14%) reported that PoC US was either not supported or allowed as a reason for its unavailability.

At sites where PoC US was available for use by EPs, nearly 50% of EPs performed PoC US and used it daily (Table 3). Only 12% of EPs used PoC US ≥80% of the time to place central venous lines. Forty-four percent of EPs performing PoC US had privileges that did not require subsequent confirmatory radiology study, and another 22% had partial privileges. Nonetheless, nearly half of EPs performing PoC US could not bill separately for use and interpretation.

## DISCUSSION

Given the growing evidence of the benefits of PoC US, it is incumbent on the emergency medicine community to identify barriers to PoC US utilization. Relying solely on the training of current residents to disseminate the use of PoC US does not address the barriers and needs of most practicing EPs, who trained prior to the widespread use of PoC US.

To our knowledge, this is the first multi-state survey to focus, at an individual ED level, on barriers to use of PoC US, a key skill for all EPs. As of 2009, only half of our sample of EDs had PoC US available in the ED. Availability differed by state, and was more common in EDs with higher volume, and EDs with a higher percentage of BC/BE EPs. These basic utilization findings are similar to those of Talley et al. from one year earlier in four different states, 6 and suggest reproducibility when this many EDs are sampled despite their location in different regions of the country. Our focus on barriers to use of PoC US builds on these confirmatory findings.

The prime reason for PoC US being unavailable or unused by EPs was lack of training. It is likely that a proportion of the 32% of respondents who did not have PoC US available or who did not use PoC US due to lack of perceived need would begin using PoC US if they had more training. Moreover, only 12% of EPs with PoC US used it more than 80% of the time to place central venous lines, which is now preferred due to its improved safety profile, 10 depicting a gap in procedural PoC US skills.

Academic centers will continue to train residents in ultrasound and recruit ultrasound fellows to grow the subspecialty. While these avenues will increase the prevalence of new EPs educated in PoC US, change will be slow if they are the sole methods the specialty relies upon. These educational methods do not address the need for many current EPs to become facile with US. Thankfully, the widespread use of asynchronous learning platforms has made it easier than ever to learn PoC US at little (if any) cost at anytime, from anywhere in the world. Education-oriented websites such as American College of Emergency Physicians’ sonoguide.com continue to grow, as do free open-access medical education forums on websites, blogs, video logs, and other Internet-based resources. 11 Moreover, several studies have highlighted that PoC US images of adequate quality can be streamed over Internet or wireless phone networks. Combined with synchronous voice or video between the examiner and educator, this enables real time education during actual scanning. In-person training will always be highly valuable though. Notably, a recent randomized trial of internal medicine interns acquiring PoC US skills via faculty-guided or self-guided curricula showed both can improve the self-reported competence of medicine interns in PoC US, but faculty-guided training was superior to self-guided training in both intern preference and skills acquisition assessed with observed structured clinical examinations. 12 In-person training, as opposed to asynchronous training, may also be more effective at improving ultrasound-guided procedural skill. Thus, for the 19% of EPs who reported PoC US availability but did not use it to place central venous lines, in-person training via skills workshops and/or distance learning with mannequins may be more appropriate.

A substantial number of the EDs in our study also reported that PoC US was not available due to high cost, demonstrating market need for low-cost devices. Previously, the American market favored high technical capability over low cost, focusing companies on full-stack devices that were function-heavy and expensive. In the case of PoC US, there are burgeoning solutions from within and outside of the United States that hope to address cost. The most expensive component of current ultrasound devices is the piezoelectric crystals or ceramics that generate and receive sound waves. Companies like Butterfly Network (www.butterflynetinc.com) are leveraging capacitive micro-machined ultrasound transducers, which have the promise of making PoC US much cheaper as well as producing better image quality. Legacy companies are also manufacturing handheld devices with fewer functions and lower cost (generally $6,000-$8,000) compared to full-stack systems. Nonetheless, these technologies continue to be expensive or under development. There is real market need to develop targeted, low-cost PoC US devices.

Finally, our data indicate that 14% of EPs reported PoC US was not supported or allowed, and nearly half of EPs performing PoC US could not bill separately for use and interpretation of PoC US. While 14% may appear low, given the convincing evidence that PoC US improves the value of emergency care, this represents a substantial number of EPs practicing within cultures that are not aligned with practice trends. EPs can advocate for adopting PoC US in their practice using the existing evidence. If EPs could generate compensation for time spent using PoC US, it would be easier for an ED to afford purchasing US equipment. Billing for PoC US can be established quickly and generate revenue to offset the cost of training and performance. These data highlight the need for EPs to advocate at their local institutions and nationally for billing parity. Nonetheless, some EDs may truly not have a need for PoC US. For example, there is likely a greater return on investment in PoC US in EDs with high patient volume that must reduce throughput times to prevent crowding and patients leaving without being seen. This cost/benefit ratio may not be favorable for EDs with lower patient volume.

## LIMITATIONS

This study has potential limitations, including the possibility of selection bias due to the specific states sampled, though consistency with the overall data from Talley et al. 6 suggests that any bias is minimal. Response bias may also affect our results. Nonetheless, we showed that response to PoC US questions in the overall sample did not vary by most ED characteristics. It is possible that data would be more accurate if measured at the level of individual EPs rather than at the level of the ED director. Yet, measuring data at the individual level – when it may reflect personal or system deficiencies – may cause individuals to falsely inflate those capabilities, obscuring the deficiencies we hoped to capture. Data acquisition at the level of the ED provides some degree of anonymity, possibly allowing respondents to be more forthcoming.

## CONCLUSION

In summary, we found that only 47% of EDs in our five-state sample had immediate access to PoC US. When access was available and PoC US was not used, the most common barriers were lack of training, lack of need (perceived or real), and high cost. There are many plausible approaches to overcome these barriers, some of which are available currently and described above. Future research should continue to define barriers as they change over time, and describe and test novel solutions to increase utilization of PoC US in emergency care.

## Figures and Tables

**Table 1:** Availability of point-of-care ultrasound in five states (n=242 emergency departments).

	Total	Point-of-care ultrasound			P-value
	
	n	No n (%)	Yes n (%)	Unknown n (%)	PoC US available Yes vs. No
Total	242	88 (36)	113 (47)	41 (17)	
State					
Arkansas	61	37 (60)	21 (34)	3 (5)	0.002
Hawaii	23	7 (30)	16 (70)	0 (0)	
Minnesota	119	33 (28)	54 (45)	32 (27)	
Vermont	13	2 (15)	11 (85)	0 (0)	
Wyoming	26	9 (35)	11 (42)	6 (23)	
Urban/rural status					
Urban	77	20 (26)	45 (58)	12 (16)	0.04
Rural, adjacent to urban	102	25 (40)	27 (43)	11 (18)	
Rural, not adjacent to urban	63	43 (42)	41 (40)	18 (18)	
ED visit volume (patients/hour)					
<1	124	60 (48)	38 (31)	26 (21)	<0.001
1.0 to less than 2.0	52	17 (33)	27 (52)	8 (15)	
2.0 to less than 3.0	23	6 (26)	17 (74)	0 (0)	
≥3	43	5 (12)	31 (72)	7 (17)	
Admission rate					
0 to less than 10%	27	18 (67)	9 (33)	0 (0)	0.02
10 to less than 20%	95	37 (39)	58 (61)	0 (0)	
≥20%	55	20 (36)	34 (62)	1 (2)	
Unknown	65	13 (20)	12 (19)	40 (62)	
Number of physician FTEs					
0 to less than 5	76	49 (64)	26 (34)	1 (1)	<0.001
5 to less than 10	58	19 (33)	39 (67)	0 (0)	
≥10	40	7 (18)	32 (80)	1 (3)	
Unknown	68	13 (19)	16 (24)	39 (57)	
EM BC/BE physicians					
0% to less than 21%	83	49 (59)	33 (40)	1 (1)	<0.001
21% to less than 80%	26	10 (39)	16 (62)	0 (0)	
≥80%	61	11 (18)	49 (80)	1 (2)	
Unknown	72	18 (25)	15 (21)	39 (54)	
Uninsured or self-pay					
0% to less than 16%	80	29 (36)	51 (64)	0 (0)	0.08
16% to less than 30%	44	22 (50)	22 (50)	0 (0)	
≥30%	30	17 (57)	12 (40)	1 (3)	
Unknown	88	20 (23)	28 (32)	40 (46)	

*PoC US*, point-of-care ultrasound; *ED*, emergency department; *FTE*, full time employees; *EM BC/BE*, emergency medicine board certified/board eligible

**Table 2:** Reasons for use of point-of-care ultrasound by emergency physicians in four states (n=123 emergency departments).

	Total responses	No	Yes
	
	n	n (%)	n (%)
Is PoC US available in ED?	114	55 (48)	59 (52)
Do emergency physicians use PoC US for care?	108	62 (57)	46 (43)
Reasons for PoC US being unavailable or not used by emergency physicians			
Limited training	66	20 (30)	46 (70)
Too expensive	66	40 (61)	26 (39)
Limited need	66	45 (68)	21 (32)
Not supported/allowed	66	61 (92)	5 (8)
Other reasons	66	57 (86)	9 (14)

*PoC US*, point-of-care ultrasound; *ED*, emergency department

**Table 3:** Use patterns of point-of-care ultrasound (PoC US) in four states (n=123 emergency departments).

	n (%)
% of emergency physicians that use PoC US	44
1–20%	5 (11)
21–40%	8 (18)
41–60%	10 (23)
61–80%	6 (14)
81–100%	15 (34)
How often PoC US is used	44
Daily	22 (50)
At least once per week	13 (30)
At least once per month	5 (11)
Less than once per month	4 (9)
% of all central venous lines placed using PoC US	42
0%	8 (19)
1–20%	12 (29)
21–40%	6 (14)
41–60%	7 (17)
61–80%	4 (10)
81–100%	5 (12)
Emergency physicians have PoC US “privileges” not requiring confirmatory radiology study	41
No	14 (34)
Yes	18 (44)
Partial/in progress	9 (22)
Emergency physicians can bill separately for use and interpretation of PoC US	41
No	20 (49)
Yes	14 (34)
Partial/in progress	7 (17)
